# Special Issue “Advances in Genome Regulation in Cancer”

**DOI:** 10.3390/ijms241914567

**Published:** 2023-09-26

**Authors:** Jekaterina Erenpreisa, Alessandro Giuliani, Mark Steven Cragg

**Affiliations:** 1 Cancer Research Division, Latvian Biomedicine Research and Study Centre, LV-1067 Riga, Latvia; 2 Environment and Health Department, Istituto Superiore di Sanità, 00161 Rome, Italy; alessandro.giuliani@iss.it; 3 Centre for Cancer Immunology, Faculty of Medicine, University of Southampton, Southampton SO16 6YD, UK; m.s.cragg@soton.ac.uk

Cancer is globally increasing. Since 2010 it has risen in incidence by 26.3%, with mortality growing by 20.9% and cancer-associated invalidity increasing by 16.0% [1], painfully demonstrating our ongoing inability to win the “war on cancer” as proclaimed by US President Nixon in 1971. It is also sobering to acknowledge in the post-genomic era that cancer is far more complex and poorly understood than previously appreciated based solely on somatic mutation theory [2]. Our clinical experience to date shows that cancer is more inherently and secondarily resistant to genotoxic, targeted, and even immune therapies [3,4] than hoped. Questions arise as to why and what can be done. Currently, there are several converging and parallel hypotheses of how the cancer genome is regulated. New ideas for therapies and various facets of these hypotheses are explored in the ten articles presented in this Special Issue [5,6,7,8,9,10,11,12,13,14].

Firstly, the astounding adaptability of cancer to survive and resist aggressive genotoxic treatments whilst at the brink of death inevitably leads us to seek understanding from the laws of regulation of complex open systems, which can resist entropy and survive at the edge of chaos through explorative adaptation and learning [15,16]. These concepts, derived from physics and then adapted to the biological realm, imply that the genome is regulated not simply by separate genes and their encoded proteins but by modules of gene networks which can dynamically rewire for adaptation to unforeseen challenges (or “stress”) and redirect gene interactive expression toward low-energy networks (attractors). These “modules” can be seen as integrated supra-molecular entities in charge of accomplishing a “high-level” function. In [6], the authors define such modules as “Supramolecular Organizing Centers” (SMOCs), a term that was coined in 2014 [17].

The exploration of these functional modules by proving the existence of a network of coherent expression in clustered assemblies of their genes is an important contribution of bioinformatics to cancer research. Complex network analytical tools allow the detection of the most relevant nodes of the networks (hubs, high-centrality nodes) for signal processing. Complex network approaches are used here in several articles [7,10,12,13,14]. Moreover, this network approach provides a new paradigm for drug discovery [18].

For better cancer comprehension and realization of this new cancer drug discovery strategy, it is important to understand if stress-induced cancer genome attractors are assembled de novo or preprogrammed during evolution. The atavistic cancer theory as a series of atavistic reversions towards a quasi-unicellular phenotype [19] states that cancer attractors were acquired early in the evolution of unicellular organisms and the transition to multicellularity. Knowledge of human gene phylostratigraphy along the 3.9 billion years of the evolution of life, from Prokaryotes to humans, provides us with the ability to identify such cancer attractors [20]. However, which genomic features (neglecting mutated genes) can define them?

The bioinformatic study of Anatskaya and Vinogradov [7] proposed to answer this question: “Polyploidy and Myc proto-oncogenes promote stress adaptation via epigenetic plasticity and gene regulatory network rewiring”. In this and another article by these authors which appeared in the same journal later [21], the authors hypothesise that stress adaptability in cancer is manifested in epigenetic plasticity, associated with traits of stemness, unicellularity, flexible energy metabolism, and a complex system of DNA damage protection. The authors show the centrality of the unicellular core of the human genome and the shift towards this ancient unicellular core through cancer cell polyploidy and formulate the “Unicellular Attractor model of cancer”. These results support the views proposing cancer’s evolutionary origin from the Amoeba (or rather Entamoeba)-like reproductive cycles, including cycling polyploidy [22].

Energy provision is addressed by Kasperski et al. in this Issue: “Life Entrapped in a Network of Atavistic Attractors: How to Find a Rescue?” [5]. Kasperski raised the important issue of the bio-energetic aspects of cancer atavistic evolution. This is a crucial point that must be addressed in order to create a coherent ‘cancer theory’ able to accommodate features like the non-mutational origin of many cancers [23,24] and the centrality of the Warburg effect [25]. In his work [5], Kasperski, similarly to the gradual atavistic reversions postulated by Lineweaver et al. [19], proposes a multi-layered model of the co-existence of bio-energetic ‘attractors’ (considered as a global control) with atavistic (more evolutionarily ancient) attractors acting as the core of a hierarchy whose ‘periphery’ is made of more recent multi-cellular adaptations. This structure implies that atavistic unicellular metabolic attractors, even if latent, are more robust and consequently more difficult to eradicate. The case of energy metabolism (and the consequent acidic nature of the cancer microenvironment) is probably the most striking example of the ‘robust’ atavistic modes that are common to all cancers, and play a crucial role in cancer development [26]. According to this view, addressing the acidic tumour microenvironment should be placed centre stage for therapeutic intervention.

In previous times, the ability of cancer to endlessly proliferate was considered its main feature, with the suggestion that inducing apoptosis and/or stopping proliferation (which are both molecularly linked [27,28]) would suffice for treatment. Contrary to this largely unsuccessful approach, two articles in this issue by Loftus et al. [6] and Egorshina et al. [9] evaluate the vulnerability of cancer to cell death mechanisms not directly linked to proliferation, such as necroptosis and ferroptosis, as possible novel strategies against cancer. In particular, Loftus et al. [6] also highlight that the cell-cycle-independent death programs are immunogenic, potentially licensing host immunity for additional antitumor activity. Identifying cell-cycle-independent vulnerabilities of cancer is critical for developing alternative strategies that can overcome therapeutic resistance. In the article [9], so-called “mitotic catastrophe” is discussed as a starting point for this vulnerability potential. Interestingly, the intrigue around “mitotic catastrophe” (where cells reach metaphase but either die or “slip” from it and can return to interphase (termed “mitotic restitution” or more recently “mitotic slippage”) and survive as polyploids, giving rise to de-polyploidised progeny) began two decades ago. The first review from the labs of Erenpreisa and Cragg, provocatively titled “Mitotic death: a mechanism of survival?” [29] with the schematic reproduced in Figure 1, was followed by a review from the lab of Boris Zhyvotovsky [30], which considered mitotic catastrophe as a “pre-stage” with an uncertain fate: death or survival. As has since been clarified, the uncertainty apparently lies in the cellular senescence of G2M- and M-arrested cells which support active metabolism by employing autophagy, underlined in the current Issue by Egorshina et al. [9] and showing that suppression of autophagy added to conventional chemotherapy can kill cancer cells by necroptosis.

In a somewhat similar approach, Cuccu et al. [12] selected non-proliferating “quiescent” cells from tumour spheres of lung and colon cancer, which were shown to be enriched with coherent stemness and EMT modules, while the proliferating cells lacked these resources of cancer relapse. Complex network analysis is at the basis of Cuccu et al.’s [12] paper, in order to both discriminate the “quiescent state” from the “proliferative state” of cancer stem cells and to identify the most promising molecular targets in terms of maximum “network centrality”.

The various cell-fate decisions associated with accelerated cellular senescence (ACS) which interrupt cell divisions, observed in various cancer cell lines treated with different genotoxic agents, was discussed in the article from the lab of Prof Ewa Sikora [10]. As already known, senescence allows cells to enter a temporary state of dormancy that eventually facilitates disease recurrence, often in a more aggressive state promoting cancer stemness [31,32]. In this case, [10], p53-wild type MCF-7 cells treated with Topoisomerase I inhibitor irinotecan were examined and shown to undergo senescence/polyploidisation through mitotic slippage, returning partially to the proliferative state from day 8 and with upregulation of genes related to the meiotic cell cycle, spermatogenesis, and EMT. These observations give credibility to the gametogenic (embryonic) theory of cancer revived from the 19th century, but with additional consideration of the polyploid giant cancer cell (PGCC) as a quasi-blastula [33,34].

Besides this, it is worth noting that these spermatogenesis-stimulating genes belong mostly to the MAGEA family of cancer–testis antigens (CTA) and are of late evolutionary origin, also known as strong oncogenes. The finding of meiotic and CTA genes in [10] is relevant to the study of Vainshelbaum et al. [13] in this Issue, in which 1474 gametogenic genes were subjected to phylostratigraphic analysis, taking all human genome genes as a reference. Several evolutionary peaks of reproduction processes were revealed. The biggest occurred in unicellular organisms, which have already been related to DNA repair, meiosis, and gametes, and was enhanced in the cancer genome atlas (TCGA) by polyploidy, thus supporting the atavistic theory of unicellular cancer attractors and the life-cycle theory of cancer via reversible polyploidy [35]. The second, smaller peak embraced the origin of embryonic development and primordial germ cells in Metazoa/Eumetazoa; it tended to fuse with the unicellular peak through activity in the polyploidy of bivalent genes [7,13]. However, it appears that two late evolutionary splashes of CTA genes preceding the evolution of hominids deviate from the otherwise slender unicellular theory of human cancer.

A study by Vainshelbaum et al. [13] showed that the reproductive modules accumulated in the human genome during the evolution of life are not only interconnected but also strongly modified by the latest CTA acquisitions in mammals and hominids. The additional CTA attractors used for stress protection in spermatogenesis were introduced in mammals with evolution of their large and developed brains through the aid of retrotransposons, along with a risk of gonadal and somatic cancer, in a process that is still not finished [36].

A large role of the domestication of retrotransposons involved in mammalian evolution is considered in the article by Lavia et al. [8], which proposes that cancer onset and progression are determined by a stress-responsive epigenetic mechanism that results from the convergence of upregulation of long interspersed nuclear element 1 (LINE-1), the largest and youngest family of human retrotransposons, providing new context for the understanding of cancer. Lavia et al. postulate that upregulated expression of LINE-1 retrotransposons and their protein products have a key role in genome damage, nuclear lamina fragmentation (a mark of cellular senescence), chromatin remodelling, genome reprogramming, and autophagy activation, yielding an increased plasticity of the nuclear architecture with the ensuing reprogramming of global gene expression, including the re-activation of embryonic transcription profiles.

With regards the embryonic nature of cancer exhibited by PGCCs as supported by the TCGA database in [13] and previous studies [33,34], the most recent paper in this Issue by Salmina et al. [14] added an unexpected aspect to the characteristics of PGCCs. As created by repeated mitotic slippage in a breast cancer cell line in response to doxorubicin treatment, the transcriptome analysis of the resulting PGCCs revealed not only female meiosis but also stark upregulation of the invasive “female pregnancy” Gene Ontology module elaborated in the innate immunity network. Correspondingly, immunofluorescence detected the markers of oocyte maturation and trophoblastic differentiation in the same PGCCs. These findings may shed new light on the metastatic process, possibly hijacking the evolutionary program of embryo implantation.

Considering the crucial role of PGCCs in tumour growth and resistance, one essential question requires attention. From the schematic in Figure 1, it can be seen that the main mechanisms of cancer resistance to treatment were already outlined twenty-two years ago: adaptation of DNA damage checkpoints in the cell cycle, resulting in polyploidy which provides the dual potential for cell death or adaptive rescue, and subsequent reduction divisions, proposed to involve recombination.

This reduction from polyploidy in somatic cancer (often described as amitotic budding, but which might better be described as coenocytosis—a postponed cellularisation after karyotomy)—remains of significant interest in cancer research. It potentially provides the basis for correction of the loss of heterozygosity (LOH) of aneupolyploid cells, resolving the so-called “Aneuploidy Paradox” and ”Mitchel’s ratchet” that would inevitably lead any aneupolyploid proliferating population to terminal decline [37]. This issue would seem to be only capable of resolution through DNA homologous recombination and is usually ignored, but the question remains—by which means does the recombination occur? Mitotic, i.e., recombination between sister chromatids; meiotic, i.e., recombination between homologues; or some non-trivial intermediate/novel pathway available to cancer cells?

Two potential answers are provided by a population geneticist Marco Archetti who in one article showed that for decreasing LOH the asexual reproduction with polyploidy and inverted meiosis can replace sexual reproduction but not with conventional meiosis [38]; in support, the signs of inverted meiosis can be found in cancer [37,39]. In the current Issue, Archetti [11] provides data showing that adaptation against LOH in cancer can be also provided by allogeneic cell fusion, a kind of polyploidisation occasionally seen in tumours. Although it cannot be ruled out that spontaneous cell fusions can take place in a small proportion of cells as a mechanism of parasexual recombination in tumour cell populations [40], the bulk of evidence presented in three articles in this Issue [10,13,14] and many others before (e.g., [41,42]) testifies to a very large cohort of activated cancer genes (hundreds) involved in meiosis and evolutionary pre-programmed gametogenesis. It suggests that gametogenic development through the soma-to-germ transition is a cancer mainstream, particularly employing mitotic slippage and resulting stress-adaptive polyploid cells [39].

In summary, these ten articles serve to highlight the complexity and increasing knowledge around how the cancer genome is regulated. Although the war on cancer has so far been lost, it is hoped that with these new insights and our increasing appreciation and implementation of systems biology that we might be able to turn the tide in the coming decades.

## Figures and Tables

**Figure 1:**
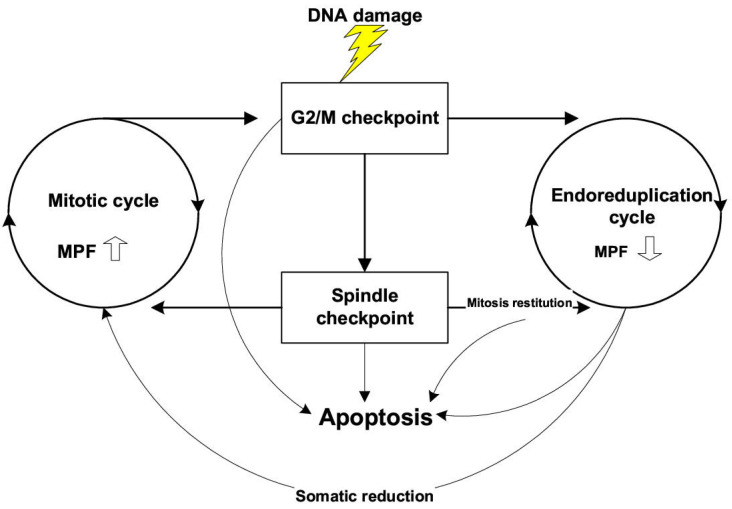
Available switch points from the mitotic pathway to the endocycle in p53 mutant cells and stimulation of this switch by DNA damage. MPF—Mitosis-Promoting Factor. Republished from [29] licensed by BioMed Central Ltd.
